# Effection of Lactic Acid Dissociation on Swelling-Based Short-Chain Fatty Acid Vesicles Nano-Delivery

**DOI:** 10.3390/foods11111630

**Published:** 2022-05-31

**Authors:** Lichun Chen, Huimin Zhao, Songwen Xue, Kexian Chen, Yue Zhang

**Affiliations:** College of Food & Biology Engineering, Zhejiang Gongshang University, Hangzhou 310035, China; m794421833@163.com (H.Z.); xuesw2564893672@163.com (S.X.); kxchem@zjgsu.edu.cn (K.C.); zhangyue@zjgsu.edu.cn (Y.Z.)

**Keywords:** fatty acids, lactic acid environment, vesicle membrane, hydrogen ions

## Abstract

Functionalized small-molecule assemblies can exhibit nano-delivery properties that significantly improve the bioavailability of bioactive molecules. This study explored the self-assembly of short-chain fatty acids (FA, Cn < 8) to form novel biomimetic nanovesicles as delivery systems. Lactic acid is involved in the regulation of multiple signaling pathways in cancer metabolism, and the dissociation of lactic acid (LA) is used to regulate the delivery effect of short-chain fatty acid vesicles. The study showed that the dissociation of lactic acid caused pH changes in the solution environment inducing hydrogen ion permeability leading to rapid osmotic expansion and shape transformation of FA vesicles. The intrinsic features of FA vesicle formation in the LA environment accompanied by hydrogen ion fluctuations, and the appearance of nearly spherical vesicles were investigated by transmission electron microscopy (TEM) and Fourier Transform Infrared Spectroscopy (FTIR). Compared with the vesicle membrane built by surfactants, the FA/LA composite system showed higher permeability and led to better membrane stability and rigidity. Finally, membrane potential studies with the IEC cell model demonstrate that lactate dissociation capacity can effectively increase the cellular adsorption of FA vesicles. Altogether, these results prove that FA vesicles can function as a stand-alone delivery system and also serve as potential development strategies for applications in a lactate environment.

## 1. Introduction

Previously, tremendous efforts have been made to develop better, more complex delivery strategies with various intelligent nano-delivery systems. Nano-assembly provides a reliable and practical approach to constructing functional assemblies through different mechanisms [1]. Small-molecule assemblies are nanoparticles (NPs) or biofilms assembled from low-molecular-weight organic compounds, and the self-assembly process tends to equilibrate to a minimum-energy tissue configuration with lower entropy than isolated monomers [2]. It has been shown to be driven by electrostatic hydrophobic interactions, which significantly improve solubility and bioavailability [3]. Studies showing vesicle morphology similar to biofilms have been used as simplified models or biomimetic studies [4,5,6]. Owing to the structural similarity of transferosomes formed with cell vesicles or exocytotic vesicles, these nanocarriers are suitable for targeted drug delivery and considered to be good options for transporting active substances [7]. Recently, vesicles as novel delivery vehicles have been widely used in various biomedical and functional food with high-loading capacity and an intracellular microenvironment response [8].

Lactic acid is an interorgan carbon source and signaling molecule that acts as a paracrine metabolic regulator to enhance crosstalk and interactions [9]. Especially in a tumor microenvironment (TME), altered glucose metabolism is characterized by enhanced lactate generation with a decrease in pH value and an increase in hypoxia [10,11,12]. Early studies of the immunosuppressive environment identified that extracellular lactate supports the generation of suppressive macrophages and regulatory T cells [13,14]. Further, lactate secretion is proton-coupled, leading to extracellular acidosis suppressing effector reduction [15]. Recent reports have highlighted that PPAR signaling activated by endogenous fatty acids contributed to immunosuppression in the TME [16]. In addition, tumor cells release greater amounts of fatty acids into the environment than normal tissues. As a result, fatty acids also affect membrane proliferation by increasing oxygen level uptake to meet nutrient utilization demands for membrane synthesis [17]. Compared with traditional delivery systems, nanoparticles with unique physical properties can efficiently penetrate and extend the time of drugs in the TME. Further elucidation of the changes in the nanoparticle structure of lactic acid at different stages in the microenvironment is required in order to design a more personalized delivery.

Despite there being a class of surfactants suitable for vesicle formation in aqueous solutions, some critical properties such as polydispersity, vesicle stability, and size still need to be considered, which are mainly related to the destructive effects of intermolecular interactions [18]. To date, there has been quite a lot of theoretical research focused on the mechanical properties of the lipid vesicular system [19,20]. As permeable vesicles, the hydrophobic core of the membrane poses a barrier to the permeation of water and water-soluble molecules into and out of the vesicle [21]. It is generally assumed that the vesicles have osmotic swelling that originates from the membrane elasticity [22], and that the swelling vesicles are due to the Donnan effect driven by solute influx [23]. Previous work by Koslov and Markin indicated that the response of submicron-sized vesicles to osmotic stress may be a characteristic quality of the expansion–burst cycle, as continuous transient pores are formed [24]. First, as solute molecules enter, the vesicle gradually expands, approaching a spherical shape [25], inflating as a sphere, and eventually rupturing [26,27]. Consequently, their volume quickly re-adjusts through osmosis to change the number of enclosed solute molecules [18,28]. Moreover, experimental studies have focused on physicochemical properties and phase transition. Matteo Gabba et al. constructed a theoretical model of the vesicle dynamics upon osmotic perturbation to describe weak acid or base permeation across the membrane of both artificial vesicles and living cells [29]. Recently, Hailong reported an artificial organelle with precisely controlled adaptive dynamic behavior in pH buffer [30]. As previously mentioned, lactate has a multifaceted role in immunosuppression and is a promising therapeutic target [31]. In addition, fatty acids form different lipid structures under solution conditions, which may affect the physicochemical parameters and stability properties of vesicles. For the perspective of rationally designing delivery systems, it is necessary to understand how the dissociation of lactic acid modifies the interaction between weak acids (lactic acid) and small molecules (fatty acids) in the microenvironment. However, evaluation of these properties has been rarely reported.

In recent years, our group has focused on the design and development of self-assembling vesicle derived from medium-chain fatty acids (MFA) and ultra-short alkylphosphonic acids [32,33]. Interestingly, the self-assembly of surfactants with small molecules can be influenced by changing molar ratio, temperature, pH and additives, maintaining their physical properties under different environmental conditions [34,35]. These characteristics empower the vesicles with the targeting abilities, moreover, their nanoscale size enables them to deliver different therapeutic approaches. In the present study, we aimed to validate the FA vesicles permeability properties in a lactic acid environment. As proof of concept, we selected a weak acid to stimulate the vesicles to compare thermodynamic stability and size distribution at different stages involved in FA vesicle swelling. We evaluated the morphological changes of lipid vesicles using transmission electron microscopy (TEM). Our results showed that the cell surface charge increases with the dissociation of lactate, resulting in the deformation of the vesicle structure and adsorption to the cell surface. We also addressed the possible use of hybrid FA/LA vesicles to effectively increase cellular adsorption in the IEC cell model, to apply the vesicles strategy to co-delivery of a wide range of drugs or develop nano-delivery of bioactive ingredients.

## 2. Materials and Methods

### 2.1. Chemicals and Vesicle Preparation

The heptanoic acid (Hep-A, C7 > 98%), lactic acid, and Di-8-ANEPPS (4-(2-[6-(dioctylamino)-2-naphthalenyl] ethenyl)-1-(3-sulfopropyl)-pyridinium) were supplied by Sigma-Aldrich (St. Louis, MO, USA). The buffer solution was prepared in deionized water. All chemicals were used as received without further purification.

Salt-free FA vesicles were prepared with ultra-pure water from the Millipore Milli-Q system (Darmstadt, Germany). Samples of the FA/LA vesicles for phase behavior were prepared by mixing individual fatty acid and lactic acid solutions.

### 2.2. Dynamic Light Scattering (DLS) and Zeta-Potential Measurements

DLS and zeta potential measurements were performed on a Malvern Zetasizer Nano ZS90 (Malvern, UK). The size was determined in the standard cumulant analysis, while the size distribution (PDI) analysis used the normal distribution algorithm of the Malvern DTS software; the scattering angle was 90°. All measurements were equilibrated for 20 min and performed three times at 25 °C.

### 2.3. Morphology Study of Assembled Structures

The morphology of the vesicles was observed with a TEM (Hitachi 7650, Tokyo, Japan) at an acceleration voltage of 120 kV and confocal laser scanning microscopy (CLSM) (Leica SP2, Leica Microsys-tems Inc., Mannheim, Germany). The sample solution (10 μL) was applied onto Holey carbon film (Quantifoil, R2/2) using a micropipette. Grids were neg-atively stained with 2% uranyl acetate. After removing the excess staining solution, the sample was dried at room temperature for TEM observation and snapshotted by an Ultrascan USC1000 2 k × 2 k camera.

CLSM images were taken to illustrate the structural changes occurring at room temperature. A drop of the sample was deposited on the glass slide surface and covered with a cover slide (Menzel-Glaser, Berlin, Germany). Two-channel image stacks were acquired in multi-track mode, using Argon lasers of wavelengths directed at the sample.

### 2.4. Calculation Dissociation Constant

The *pK_a_* values for all lactic acids in water were determined [22]. Briefly, 50 mM of each lactic acid solution was monitored with a precision pH meter at 25 ± 0.5 °C. The *pK_a_* was calculated from the pH by applying Equation (1)

(1) $$\mathrm{pH}=pK_{a}+log\frac{\left(H-H_{min}\right)}{\left(H_{max}-H\right)}$$

where, *H_max_* and *H_min_* were the maximum [H] measured at the maximum and minimum pH values of the solution, respectively. Plots of log [(*H* − *H_min_*)/(*H_max_* − *H*)] against pH were linear with the intercept equal to *pK_a_*.

### 2.5. Cell Culture and Membrane Dipole Potential

The intestinal epithelial cells (IEC) were purchased from the Chinese Academy of Science and cultured at 37 °C, in 5% CO_2_ and high glucose Dulbecco’s modified Eagle’s medium (DMEM, Sigma-Aldrich, St. Louis, MO, USA) 147 with 10% fetal bovine serum (FBS, Shanghai, China), 2 mM L-glutamine, 100 U/mL penicillin, and 100 U/mL streptomycin. Membrane dipole potential measurements were performed according to our previous method [36]. Briefly, IEC cells were seeded in 6-well plates at approximately 5 × 10^4^ cells per well. After 24 h of incubation, the cells were loaded with 5 μM Di-8-ANEPPS in assay buffer for 2 h. After washed twice with culture medium, different test samples were added. The images of the cells were captured directly using an ORCA-495 CCD camera (Leica, Mannheim, Germany; λex = 535 nm and λem = 565 nm were used to detected cells, λex = 610 nm and λem = 700 nm were used to detected liposomes). All sample images were acquired four times.

### 2.6. Infrared Spectroscopy and Computational Methods

FTIR characterization was performed using a Bruker VERTEX 70 (Bruker, Karlsruhe Germany) equipped with a KBr beam splitter. The changes in intensity of the C=O, C-O, and -OH stretching modes were focused on Attenuated Total Reflectance (ATR) mode from 800–3400 cm^−1^ region at a resolution of 4 cm^−1^.

The interactions between fatty acids and lactic acid have been carried out theoretically within the Gaussian09 suite of programs (Gaussian 09, Revision D.01; Gaussian, Inc.: Wallingford CT, USA, 2009) according to the methods reported by our group previously [32,37]. In brief, all the structures were geometrically optimized with full degree of freedom by using the hybrid functional B3LYP method. The stability of the method was verified by the vibration frequency. The intermolecular hydrogen bond interaction energies were calculated at the m06-2×/6-311 + G (D,P)/B3LYP/6-31G (D,P) level and corrected at the m06-2×/6-311 + G (D,P) level.

## 3. Results and Discussion

### 3.1. Fatty Acid Vesicular Properties

**Figure 1:**
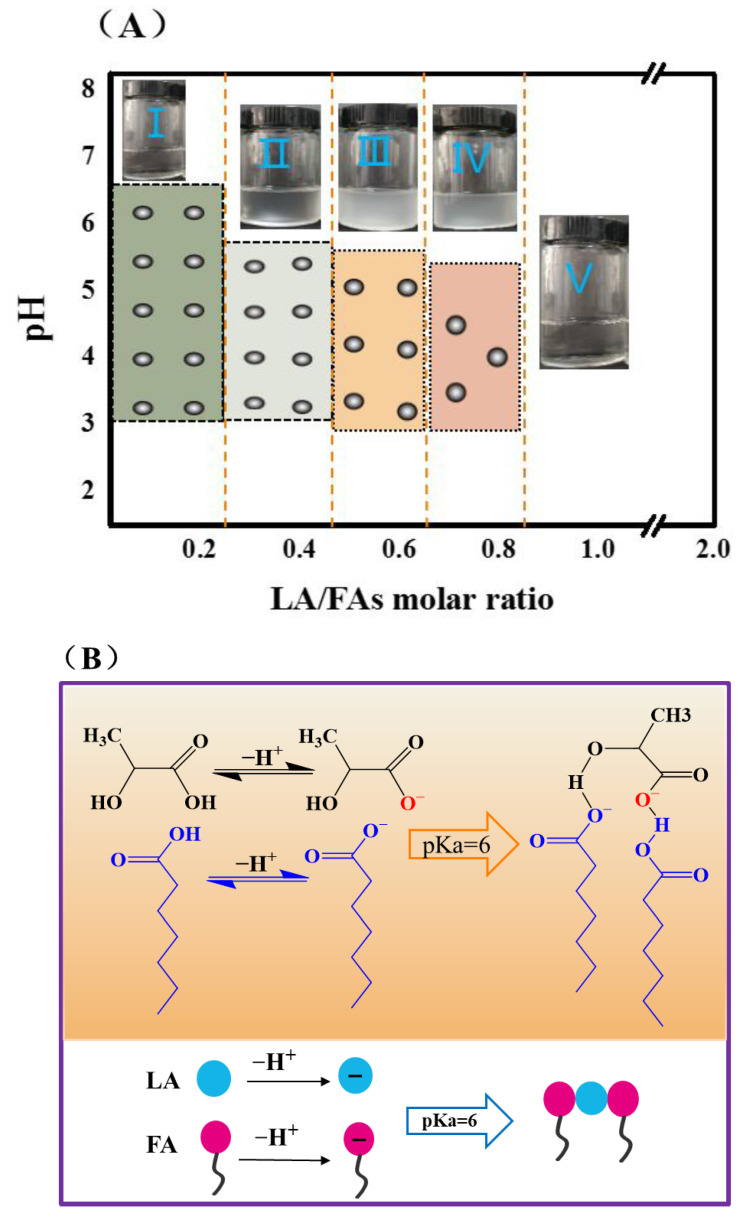
(**A**) The phase diagram of fatty acid vesicles was established as a function of pH and LA/FA molar ratio (R = LA/FA). (**B**) Expression of the vesicle formation mechanism. Lactic acid dissociates to form carboxyl group, which is connected to carboxylate ion of fatty acid through hydrogen bond under the charge interaction.

**Figure 2:**
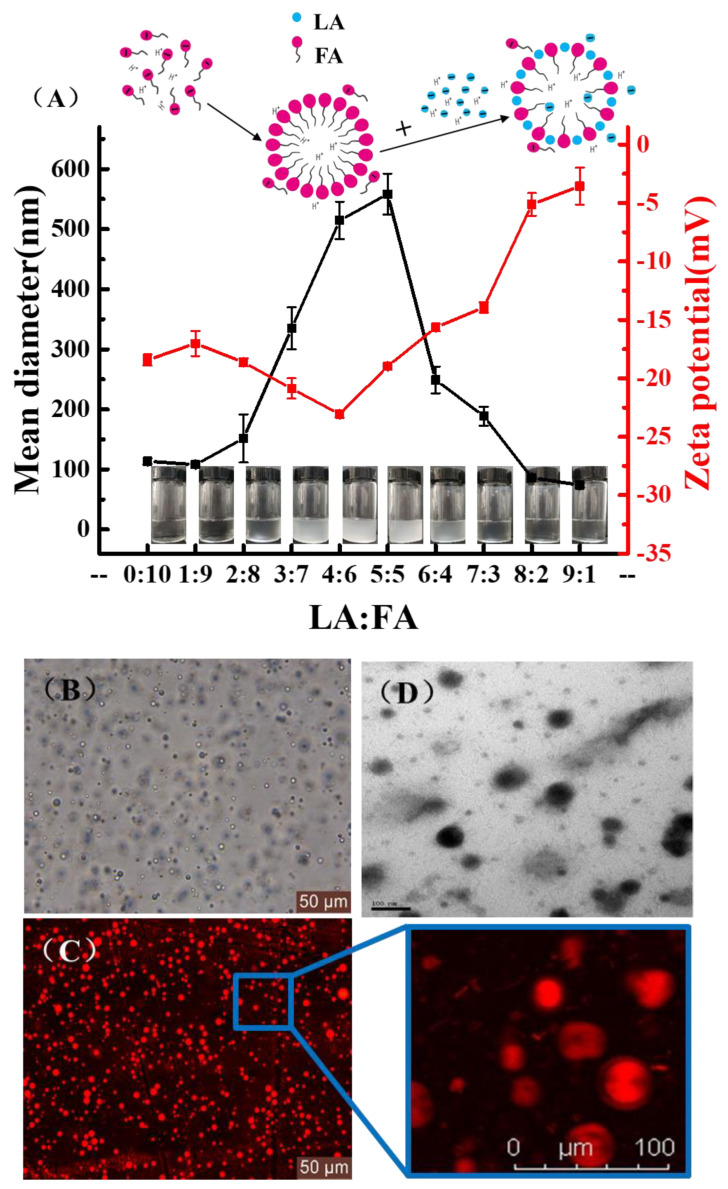
(**A**) The size and zeta-potential of FA vesicles mixed with LA at different molar ratios. (**B**) CLSM microscopic images of homogeneous vesicles were made by LA: FA (1:1) mixture. (**C**) Labeled with 1 mol% RhB. (**D**) The vesicular aggregates by TEM.

Phase behavior for the FA/LA mixtures (molar ratio R = LA/FA) was established as a function of pH and LA/FAs molar ratio (Figure 1A). In a portion of the phase diagram as the pH values were less than 3 or greater than 7, the samples were very homogeneous regardless of the molar ratio of LA/FA, and no turbid aggregates were observed by visual inspection and phase contrast microscope. Simultaneously, the apparent high-viscosity isotropic phase of the turbid solution was consistent with the formation of vesicles in the range of pH of 3–7. However, when the molar ratio reached 0.5–0.6, the number of vesicles appeared to be the highest, and the shapes were more regular. At this moment, we detected a critical micellar solution of heptanoic acid consistent with our previous report [32]. Furthermore, the formation of initially spherical vesicles was determined by confocal microscopy, and the number of vesicles changed as the solution mixing ratio was changed. The average size of FA vesicles was 300–400 nm as observed by CLSM (Figure 2B,C). However, the smaller size of 240 nm was observed for the vesicular aggregates by TEM (Figure 2D). The average hydrodynamic diameter of FA vesicles was measured by DLS to be 400–580 nm which was larger than that observed by TEM, probably arising from the momentary adhesion of vesicles.

Subsequently, we evaluated the changes in ζ-potential based on different LA/FA mixing ratios (Figure 2). Initially, with a low proportion of LA in the mixture, the vesicles acquired charges from the electrolytic environment, and the associated ζ-potential was negative (≈−25 mV), which was mainly from the dissociation of FA. It increased with the proportion of LA in the sample and reached a maximum of −4.2 mV at a 1:1 molar ratio. Notably, at mixing ratios below 40%, the addition of lactate resulted in an increase in ζ-potential from −25.2 mV to about −16.2 mV. Above this ratio, the layer reversed its charge (FA- were replaced by LA-ions) such that the ζ-potential varied with lactic acid concentration and a rapid rise was observed. At a lower LA proportion, the excess FA ions in the vesicular dispersion produced a negative electrical layer with a negative ζ-potential. Large amounts of lactic acid induced changes in the solution environment, resulting in considerable discontinuities in the dense structure of the FA vesicles, leading to primary microstructural disruption (Figure 1B), followed by the formation of large and stable composite aggregates [38].

### 3.2. The pH Causes Ion Concentration Changes

In order to study the perturbation of FA vesicles by lactate, we assumed that FA vesicles were in a semi-permeable state to evaluate the dynamic balance of water ionization in the solution environment (Figure 1B). The species distribution diagram for vesicles were calculated using Equation (2).

$$H_{2}O<=>H^{+}+OH^{-}$$

$$∆\left[H^{+}\right]={\left[H^{+}\right]}_{exp}-{\left[H^{+}\right]}_{0}$$

(2) $$C_{\left(LA^{-}\right)}=\frac{K_{a}C_{{\left(LA^{-}\right)}_{0}}\;}{C\left(H^{+}\right)+K_{a}}$$

In this equation, *C_(LA_^−^_)_* was the concentration of LA^−^ anions, *K_a_* was the LA dissociation constant. *C(H^+^)* was the concentration of hydrogen ions in solution, and *C_(LA_^−^_)0_* was the initial concentration of LA. The concentration of [H] produced by the change of pH was the most important factor for weak acid dissociation. According to the *Gibbs–Helmholtz* equation and the definition of *K_a_*, the dissociation constant (*pK_a_*), which directly reflects the dissociation ability of a compound in aqueous solution, is positively correlated with *K_a_* value [39,40]. The fact that solute molecules permeated into the vesicle implies that the vesicle progressively swells subsequently approaching a spherical shape; and its volume quickly readjusts through osmosis to change the number of enclosed solute molecules. In addition, the permeation of hydrogen ions between weak acids increases the change in ion concentration, which may swell the membrane. Therefore, the intrinsic membrane curvature resulting from a pH-dependent phase change was consistent with the expected increase in vesicle volume.

### 3.3. Changes in Cell Membrane Properties Caused by LA

**Figure 3:**
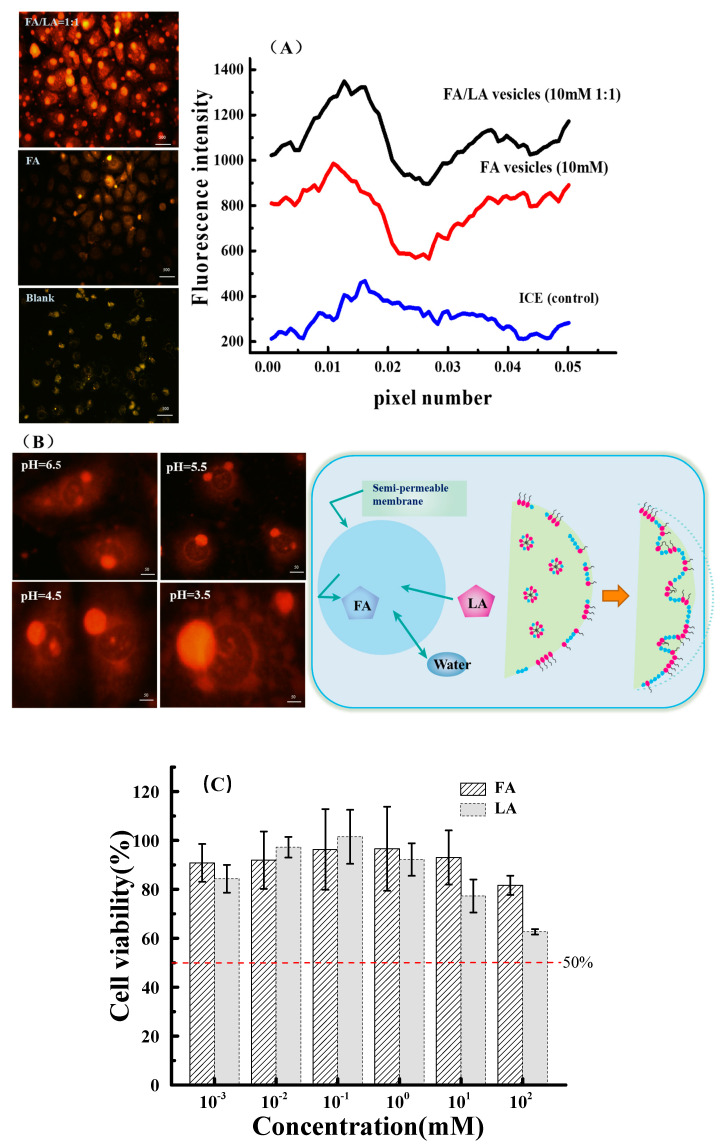
(**A**) IEC cells labeled with membrane potential fluorescent dye. The fluorescence intensity was positively correlated with the membrane potential. The addition of FA vesicles caused changes in the cell membrane potential, and the IEC membrane potential was the largest in FA/LA vesicles (R = 1:1). Scale bar: 500 μm. (**B**) The fluorescence photos of IEC at different pH with FA/LA vesicles (R = 1:1). Schematic display that the permeable properties of vesicles allow for modification of the solvent quality of the inner medium, via a phenomenon of osmosis. Arrows represent osmotic influx of solvent through the vesicle membranes and the leak-out of the inner solution through the transient pore. Scale bar: 50 μm. (**C**) The cytotoxicity of fatty acids and LA against IEC cells in vitro.

In order to understand how LA dissociation affects the osmotic swelling of FA vesicles and the interaction with cell membranes, we performed dipole potential measurements in the FA vesicle’s membranes by a dual wavelength ratiometric method using a voltage-sensitive prob. The fluorescence dye Di-8-ANEPPS is sensitive to dipole potential and to the change in the dipolar field at the membrane interface. As shown in Figure 3A, we could clearly find the effects of FA vesicles and FA/LA vesicles on the IEC cell membrane dipole potential. From the change in membrane potential, the dipole potential of the ICE cell membranes was originally 200 mV. After the addition of FA vesicles (10 mM), the membrane dipole potential exhibited a progressive increase reaching a value of 821 mV (i.e., increased by 400%). However, the membrane dipole potential increased up to 1000 mV (25% increase) in the presence of FA/LA vesicles (R = 1:1, 10 mM). Simultaneously, we also noticed that the spherical structures of vesicles were observed in both the FA vesicle and FA/LA vesicle groups (Figure 3B). At the beginning, the pH of the FA vesicle solution was stable at around 6.5, and the pH of the solution decreased continuously with the addition of lactic acid. Solution pH reached 3.5 for the FA/LA vesicles (R = 1:1, 10 mM) to form stable structures. Under lactic acid conditions, the spherical structure of FA vesicles became larger due to the pH drop caused by lactic acid dissociation accelerating the vesicle swelling. The increased permeability of the FA/LA vesicles may be explained in terms of the area expansion of membranes caused by the inclusion of surface charge density [22]. In general, the increase in area per charge accompanied with a decrease in pH implies that the membrane has become more disordered or more loosely packed. On the other hand, this increase in membrane fluidity with the addition of weak acid leads to an increase in membrane permeability in the presence of weak acid. Additionally, the vesicle membrane separated the interior volume containing an aqueous solution, which allows free exchange of water between the interior and exterior, with the flux determined by the membrane composition [41,42]. This [H] concentration induced ion concentration variety which was verified by adding lactic acid to FA vesicles with a change in pH value. By assuming charge conservation in fluid membranes, the increase of surface charge density per molecule may be associated with a decrease in lactate concentration. This is in overall agreement with the fact that the lactate microenvironment increased the surface charge accumulation of vesicles and promoted the adsorption of vesicles to the IEC cell membrane.

For biological applications, non-toxicity and biocompatibility of materials are indispensable for nano-delivery system. We examined the cytotoxicity of fatty acids and lactic acid against IEC cells in vitro. As shown by the MTT result (Figure 3C), fatty acids and LA exhibited low inhibition rates of less than 80% in terms of a concentration range up to 1 mM. We also noticed that the viability of IEC cells dropped dramatically once the concentration reached 100 mM; and calculated the half-maximal inhibitory concentration (IC50), which made inhibition more intuitive. It can be inferred that the high concentration of the acidic environment causes a rapid influx of water via osmosis into the cells, causing them to swell, rupture, and die. Our vesicles are used at concentrations well below 10 mM without any adverse effect on various cell lines.

### 3.4. Intermolecular Interaction between FA and LA

**Figure 4:**
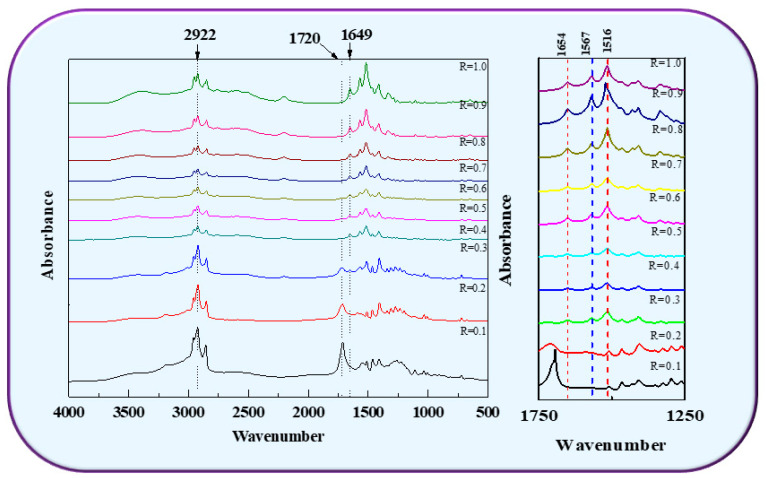
FT-IR spectra of FA/LA with different molar ratio vesicle sample.

The FTIR spectrum showed that lactic acid in the acidic environment has a protonated carboxyl group (COOH) and a deprotonated form (COO^−^). With the increasing mole ratio, the state of lactate gradually changed from COOH to COO^−^. Therefore, as shown in Figure 4, at 1734 cm^−1^ the band intensity near C=O (of fatty acid) decreased, and the intensity at 1567 cm^−1^ COO^−^ increased. When the mole ratio further increased to R = 0.9, there was a decrease of the spectral band COO^−^ at 1567 cm^−1^. The surrounding area at 1559 cm^−1^ became larger. These changes can be explained by the pH condition change in the FA/LA mixed system. When lactic acid was in the state of the protonated carboxyl group (COOH), the energy level of asymmetric stretching was like that of carboxyl (COO^−^), there could be a Fermi resonance interaction between them, and the IR intensity mode was 1567 cm^−1^ and absorption was 1647 cm^−1^ due to the stretching frequency of the COOH bond. The absorption observed at 2850 and 2922 cm^−1^ further confirmed the [COOH] stretching of fatty acids due to intermolecular hydrogen bonds.

**Figure 5:**
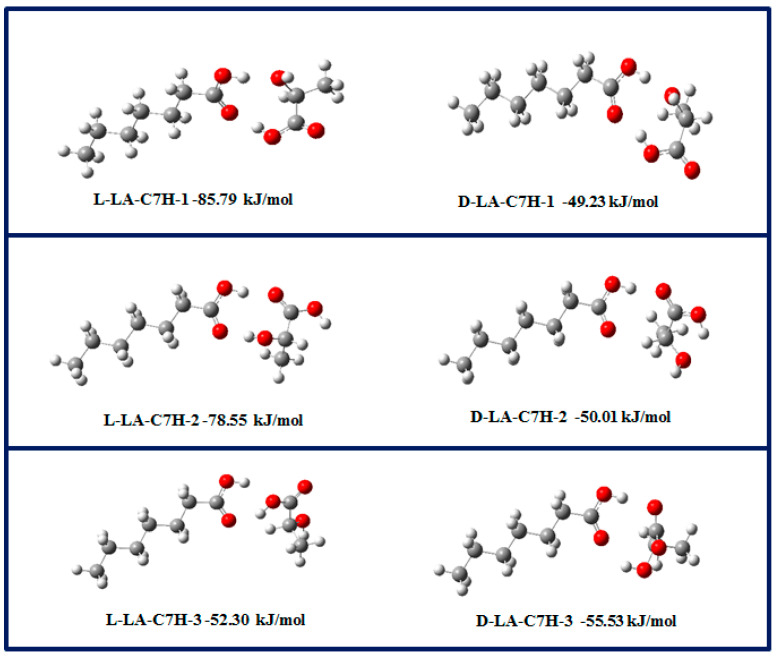
The dominant 1:1 H-bonding interaction geometries between fatty acid and L/D-LA at the B3LYP/6-31G (d, p) level along with the corresponding H-bonding energies at the M06-2X/6-311 + G(d, p)//B3LYP/6-31G(d, p) level.

In order to clarify the mechanism of the microstructure formation, we further studied the hydrogen bond interaction between fatty acids and lactic acid (D/L) by density functional theory. Three hydrogen bond conformations of heptanoic acid [cc-n (*n* = 1–3)], two hydrogen bond conformations of lactic acid [gg-n (*n* = 2)], and six hydrogen bond conformations of heptanoic acid and lactic acid [cg-n (*n* = 1–10)] were considered in the calculation. The main conformations of each type of hydrogen bond interaction are shown in Figure 5. The strongest hydrogen bond interaction lies in the interaction between the carboxyl groups of related molecules. These optimized conformations have very close hydrogen bond interaction intensities, in which D-lactic acid is larger than L-lactic acid. All these interactions were involved in the self-assembly process of the mixture of heptanoic acid and lactic acid. Therefore, it can be speculated that the hydrogen bond interaction between heptanoic acid and lactic acid (D/L) would have been dominant with the change in time of the self-assembly process. This may be due to the change of the ionic strength of the solution and the decrease of the free heptanoic acid concentration, which reduces the possibility of hydrogen bond interaction between heptanoic acid and lactic acid.

### 3.5. Weak Acid Changes the Distribution of Ions in LA/FA Vesicles System

**Figure 6:**
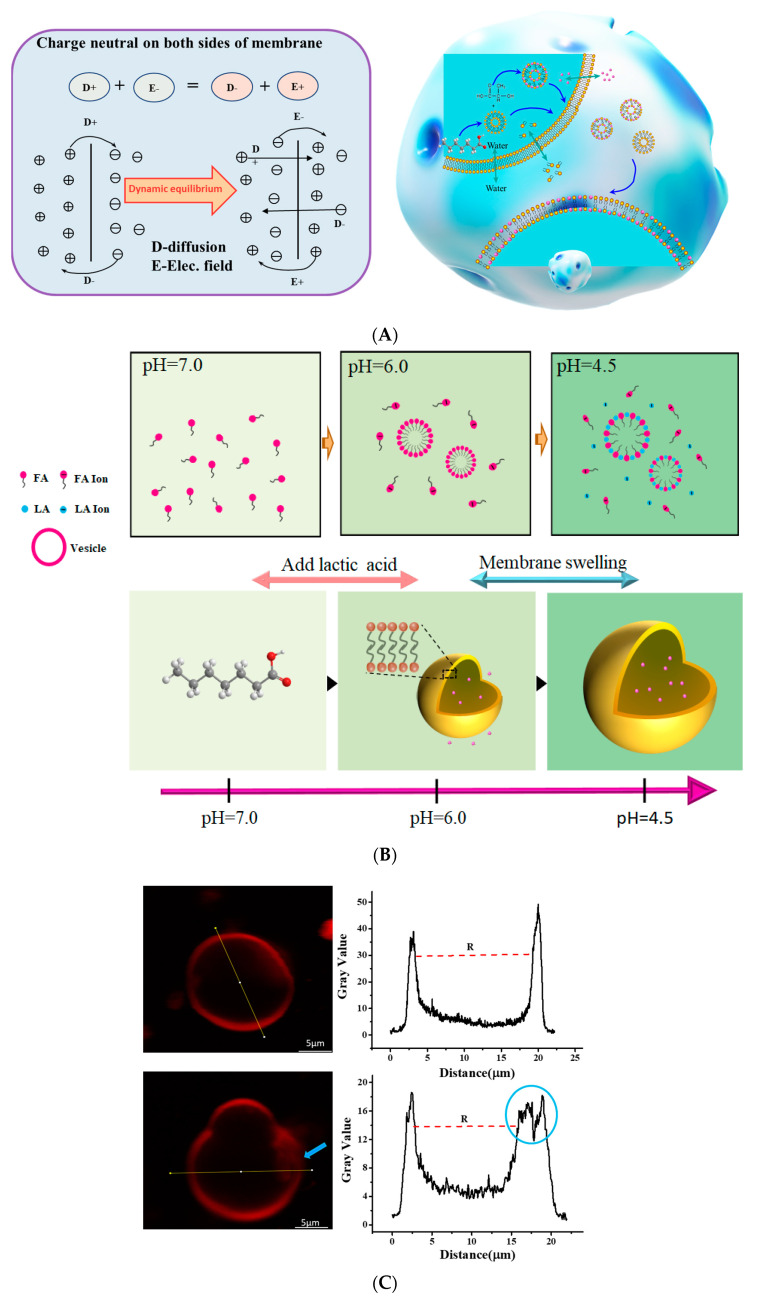
Schematic diagram of homogeneous FA vesicles exhibit swelling properties when subject to LA conditions. (**A**) The charge was neutral on both sides of semi-permeable membranes. (**B**) After the addition of LA, the system pH was changed by the dissociation of lactic acid, and gradually reassembled to LA/FA composite vesicles. (**C**) The fatty acid vesicles are labeled with fluorescent probes, and the structural changes of the vesicle membrane can be analyzed according to the fluorescence intensity. With the change of lactic acid in the solution environment, the vesicles bud and swell in radius. When the vesicle is a spherical structure, the fluorescent label on the membrane surface is the strongest, and the middle is the vesicle diameter R. When vesicles appear indented, the membrane surface fluorescence decreases to form divisions.

When a weak acid was added to the solution, the ion concentration in the solution increased with the addition of the weak acid solution concentration, and the ion distribution inside and outside the membrane was not uniform (Figure 6A). The interaction between vesicles and external ions can be analyzed and explained by the Donnan effect and Nerst–Plank equations [43,44].

(3) $${\left[H^{+}\right]}_{in}\times C_{\;{\left(LA^{-}+FA^{-}\right)}_{in}\;}={\left[H^{+}\right]}_{ex}\times C_{\;{\left(LA^{-}+FA^{-}\right)}_{ex}\;}$$

where *[H^+^]_in_* and *[H^+^]_ex_* were the concentration of hydrogen ions in and out of the vesicle, respectively. This permeation heterogeneity was caused by Donnan membrane equilibrium. It was considered that when the ion exchange membrane was immersed in the electrolyte solution, the ions in the electrolyte solution exchanged with the ions in the membrane forming an equilibrium system. The subsequent ordered structure formation was mainly driven by two intermolecular force non-polar tails and repulsive hydrophilic interactions between the molecules. The energy state of the water molecules on the hydrophobic surface was always higher and the hydrogen bonds were lost to minimize the area exposed to the water molecules. Therefore, controlling the pH value and molar ratio of the mixture were key parameters for vesicle formation and stability. Since the destruction of FA vesicles by LA began with dissociation, the spacer group was connected to the carboxylate ion by hydrogen bonds, whereas in our case, this connection was generated via charge interaction (Figure 1B). In their constant struggling with the environment, the anionic-rich side formed in the FA/LA system converted FA ions into FA/LA hydrogen bonding transitions. This subsequently raised the insertion of the latter into the FA vesicles until it became saturated and unstable as a result of the composition change of FA vesicle system.

Combining the relationship between vesicle volume and surface area explains the intermolecular forces behind molecular aggregation. Schematic diagram (Figure 6A) shows the permeable properties of vesicles allowing a change in the solvent quality of the internal medium through the phenomenon of osmosis. Furthermore, the diffusion of [H] through the shell of microcapsules composed of fatty acids triggered FA/LA vesicle assemblies, which induced fast exchanges of acid radical ionic osmoses. According to the concepts of thermodynamic equilibrium, a part of the lactic acid anion and fatty acid radical reformed new vesicles, and a part penetrated the vesicles in the process of vesicle assembly and disassembly. Furthermore, because of electrostatic repulsion, anions could permeate across the membrane to eliminate the existing concentration difference between the inside and outside of the membrane (Figure 6B). With the addition of a small amount of LA, there was almost no change in the composition of FA vesicles, and lactic acid ions entered the vesicles or were adsorbed on the membrane surface. In the osmotic stress cycle, the osmotic influx of water through the semi-permeable boundary expanded the vesicles and tightened the boundary membrane, thereby opening a tiny transient hole, releasing some internal solutes before resealing (Figure 6C). With the continuous addition of LA, the formation of FA/LA hydrogen bonds changed the membrane surface charge of the system, and the mixture could gradually form composite vesicles. The FA vesicles were subject to an osmotic attack caused by lactic acid dissociation, far away from the equilibrium, resulting in a thermodynamically unstable state, followed by the formation of complex vesicles in the new system.

A simple approach to this process is to consider a spherical aggregate, which consists of N number of molecules. If the radius of the vesicles was assumed to be 1 μm and the vesicle concentration was N = 10^6^/mL, the vesicle volume *V_lipo_* and total surface area *S_lipo_* were determined by following formula:

(4) Vlipo=(43×R3)×(N2)×V=1.256×10−5

(5) Slipo=(4πR2)×(N2)×V=9.42cm 2

Supposing that the area or volume point of an ion is approximately equal to the radius of the ion, the total surface area of vesicles *Q*_s_ [I], theoretically the maximum number and concentration of ions can be calculated as follows:

(6) $$Q_{s}\left[I\right]=S_{lipo}/\left(4\times \pi \;{\left(r\left[I\right]\right)}^{2}\times M\right)$$

(7) $$Q_{v}\left[I\right]=V_{lipo}/\left(4/3\times \pi \;{\left(r\left[I\right]\right)}^{3}\times M\right)$$

(8) $$\;C_{max}\left[I\right]=Q_{v}\left[I\right]/V_{lipo}$$

Here, the total surface area of FA/LA vesicle obtained with our correction using Equation (6) was higher than that from the FA vesicle by Equation (4), for both the total surface area and vesicle volume. Note that the usual practice is to treat the mid-plane of the vesicle layer as the membrane with zero-thickness. Nevertheless, the dependence of membrane tension on the vesicle size may serve as a mechanism for maintaining vesicle homeostasis [45]. When adding a weak acid, the FA/LA vesicle size increased during induced swelling and reached a stable status after equilibration. The ion concentration difference across the bilayer membrane increased with the swelling level, which translated to an increase in the membrane tension. The membrane curvature of lipid vesicles was caused by asymmetry, which has been previously determined by comparing the shape of the curved membrane with that of its liquid counterpart [38,46]. However, in this previous study, thinner and more disordered similar membranes were observed for smaller vesicles, which was in sharp contrast to our observations. Recent studies have shown that small molecules can be exchanged by osmosis through the perforated vesicle membrane, similar to a dialysis step. Moreover, the progressive transport of molecules from the external phase of osmoses to the inner phase could induce a pH-triggered and of multiple weak interactions between ion domains. Furthermore, the free energy appears induced by the distance within a particular ion and can change repulsive into attractive interactions between like charged surfaces.

## 4. Conclusions

In summary, in the lactic acid environment, the size and potential of the formed FA/LA vesicles were higher than those of FA vesicles formed from a single constituent fatty acid, which were 450 nm and −13.81 nm, respectively. We constructed short-chain fatty acid vesicles to verify the interaction of LA ions with FA vesicles. It was conceivable that charges in the electrostatic environment induced by a weak acid directly mediated the membrane’s permeability and flexibility. When subject to an osmotic change, the FA vesicles respond by producing swell–burst cycles. The influx of LA ion made the FA vesicles swell and therefore rendered the membrane tense, in which an interplay of permeability and deformability regulation osmotically induced vesicle deformation and reformation. Lactic acid is an important metabolic mediator of tumor–stroma interaction in TME and increased permeability of the FA vesicle membrane could be a possible mechanism for the biochemical pathways of specific molecules (for example, lactic acid). Thus, the strong interaction between a weak acid and vesicles has potentially major implications for vesicle fusion in vivo. This discovery holds promise for the development of swelling-based nano-delivery of short-chain fatty acid vesicles for the development of functional foods for cancer patients.
